# Supplementary figures and images for: Ipsilateral or contralateral boosting of mice with mRNA vaccines confers equivalent immunity and protection against a SARS-CoV-2 Omicron strain
Source: J Virol. 2024 Aug 28;98(9):e00574-24. doi: 10.1128/jvi.00574-24 (PMC11406931; doi:10.1128/jvi.00574-24)

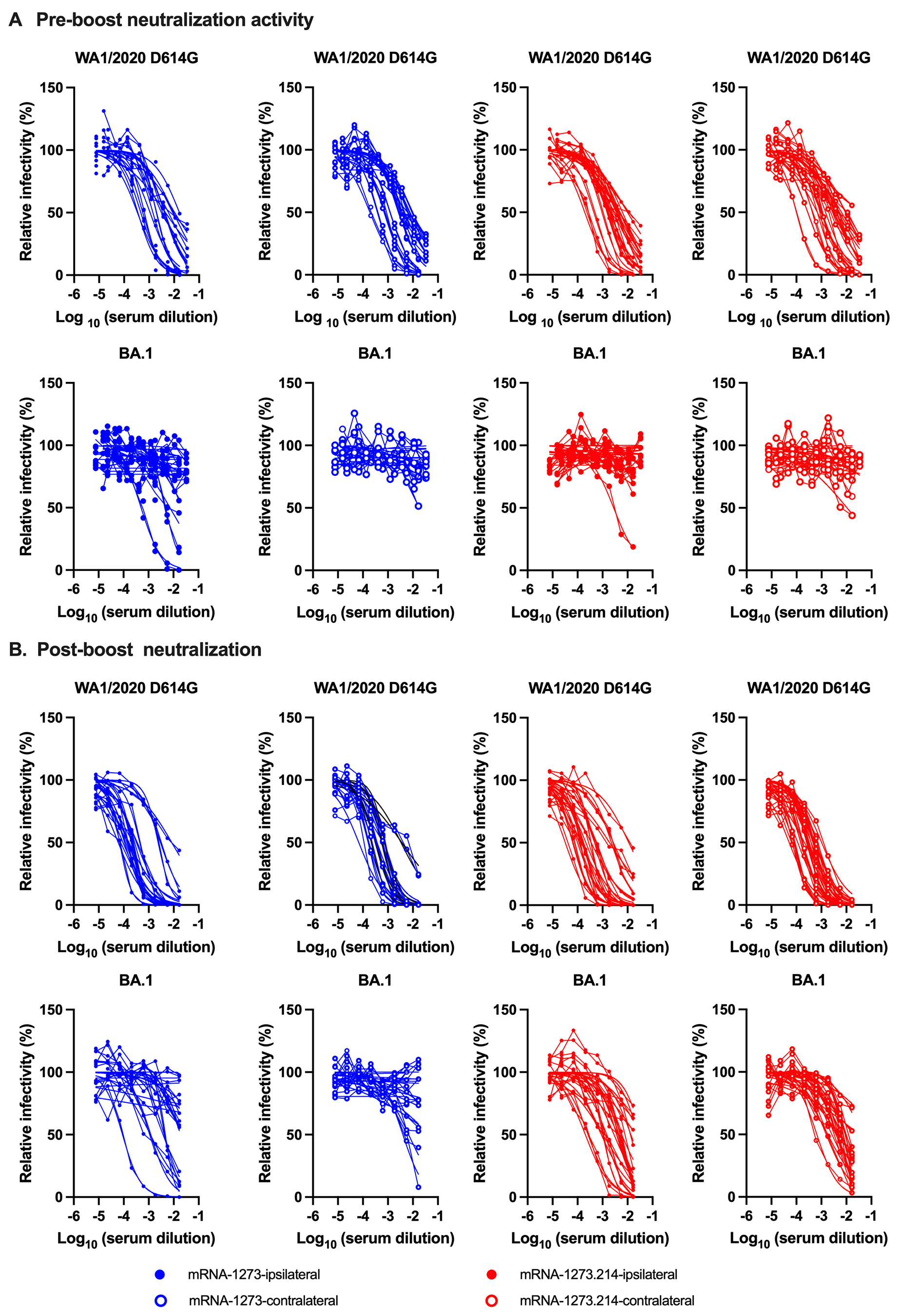

Supplement: Figure S1 — Serum neutralization of WA1/2020 D614G and BA.1 viruses. [file jvi.00574-24-s0001.tif]

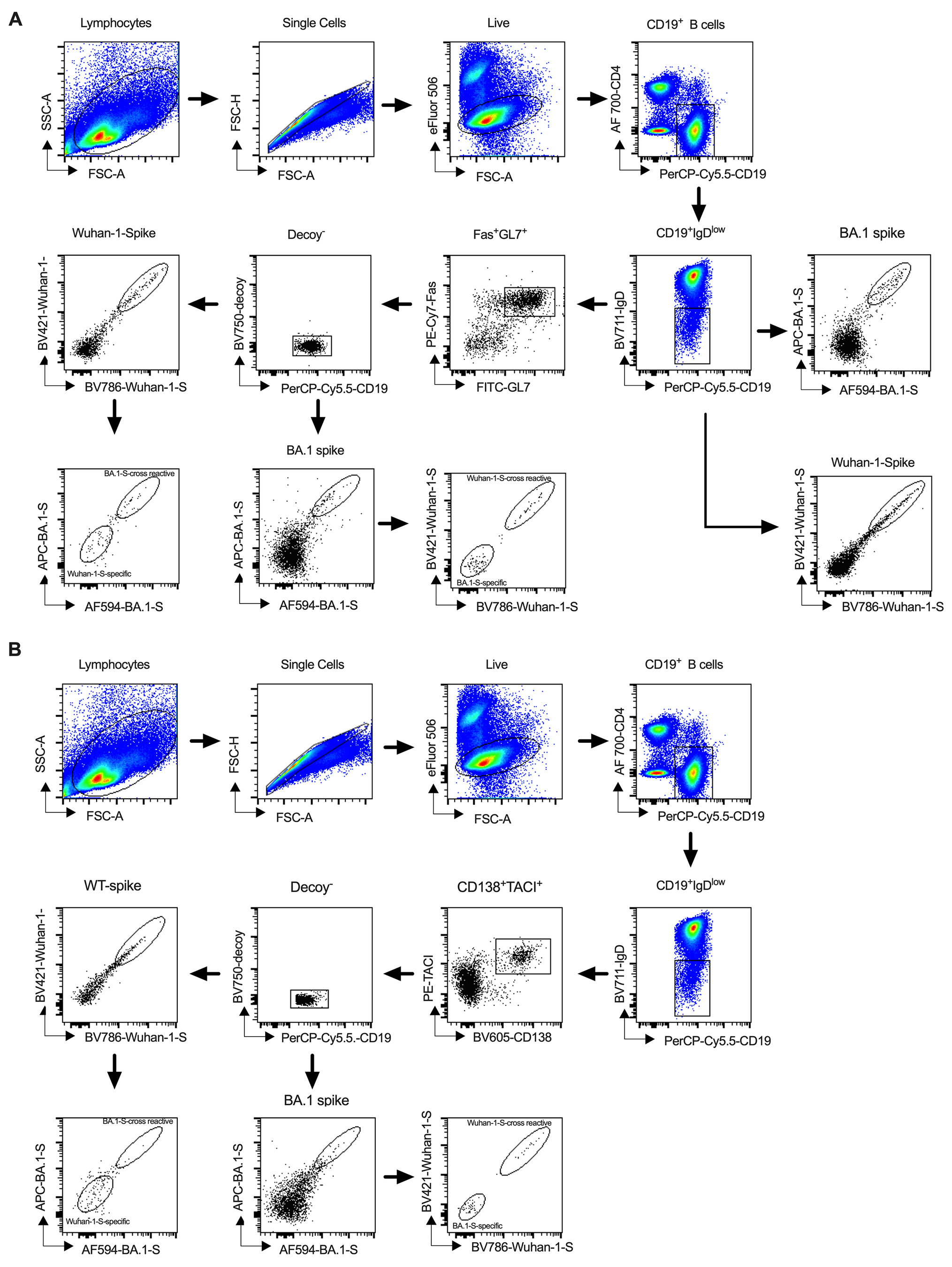

Supplement: Figure S2 — Flow cytometry gating strategies for B cells in the LN and spleen. [file jvi.00574-24-s0002.tif]

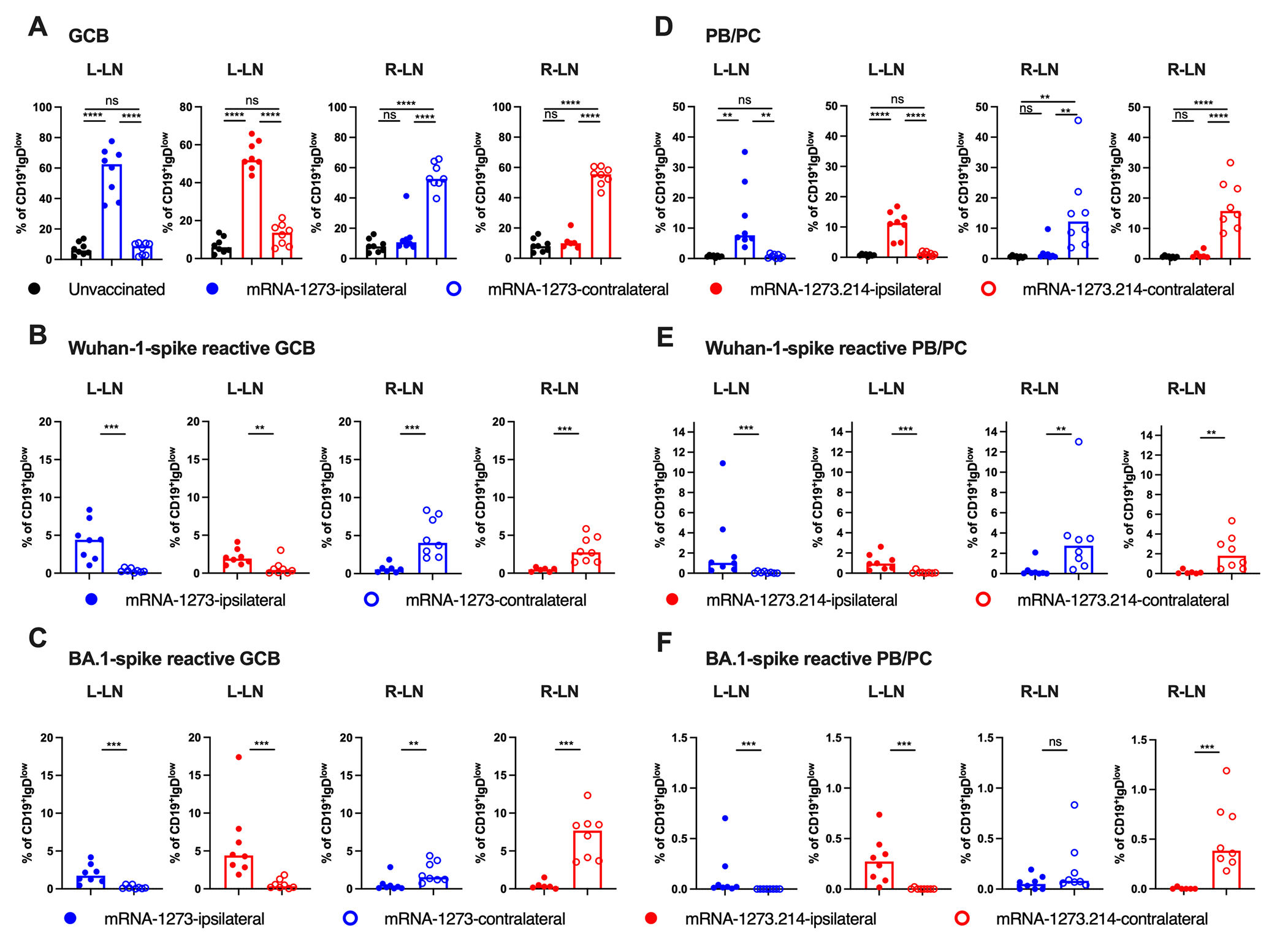

Supplement: Figure S3 — Frequency of germinal center B cells and plasmablast/plasma cells. [file jvi.00574-24-s0003.tif]

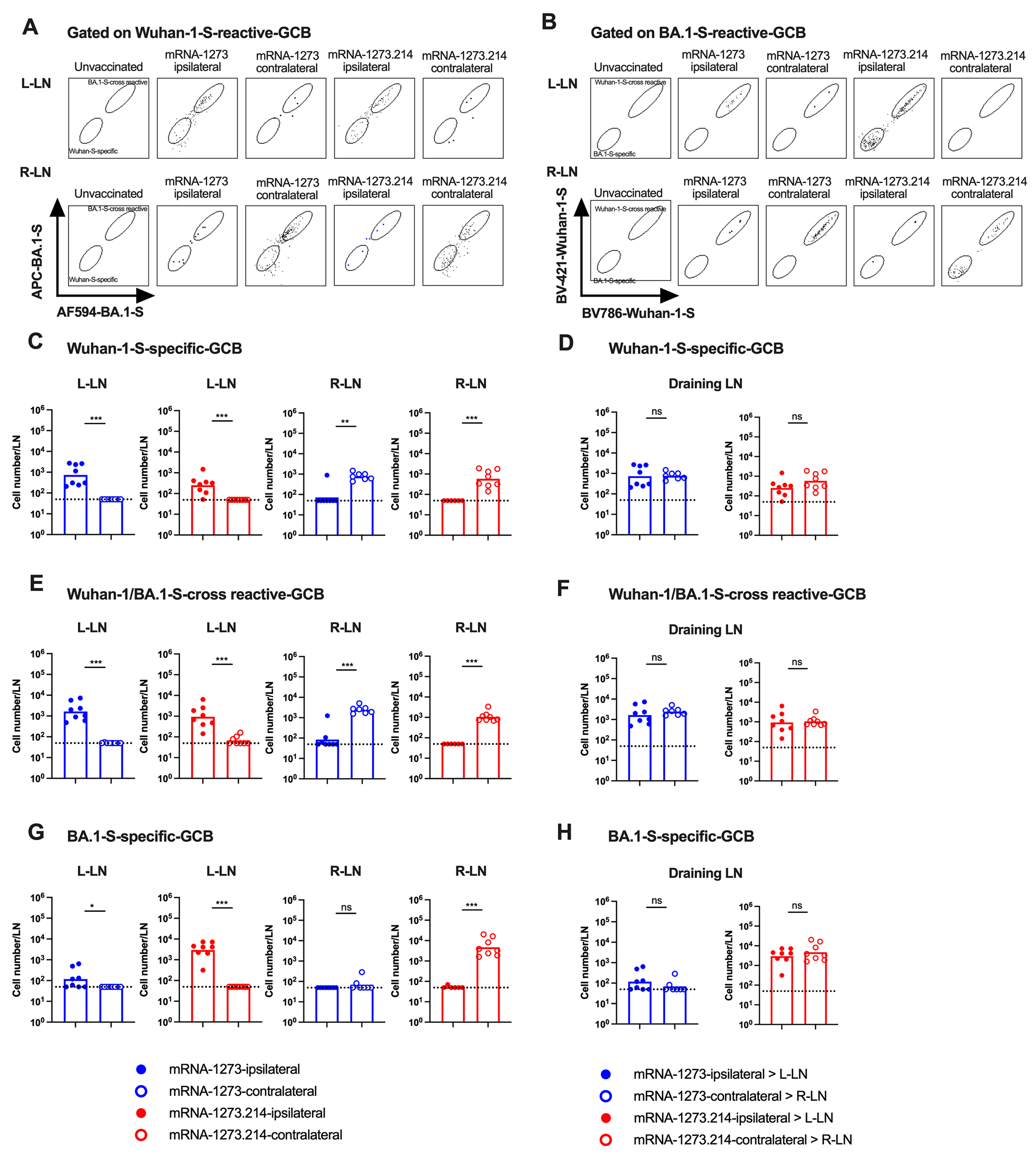

Supplement: Figure S4 — Virus type-specific and cross-reactive GCB responses in lymph node. [file jvi.00574-24-s0004.tif]

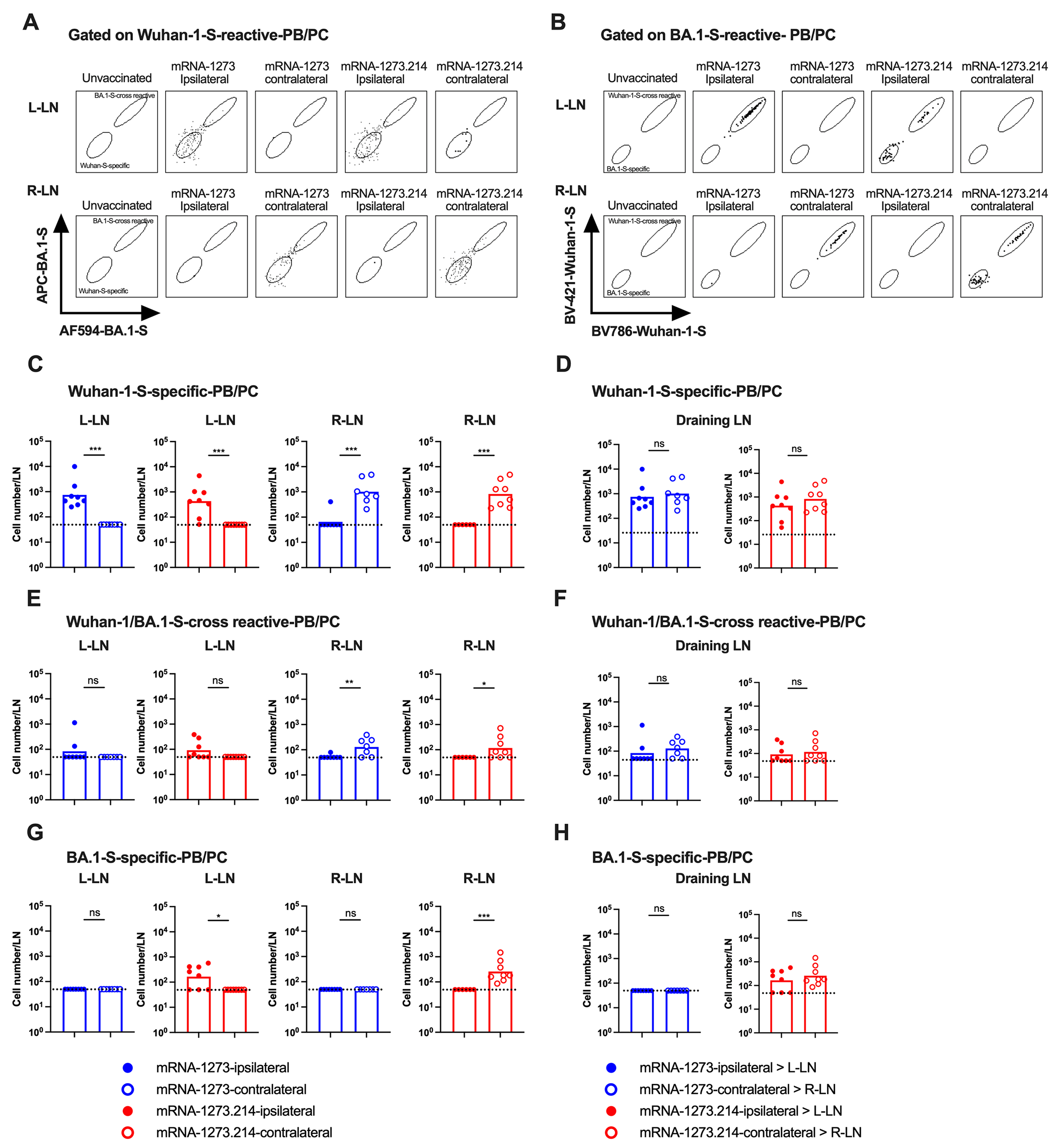

Supplement: Figure S5 — Virus type-specific and cross-reactive PB/PC responses in lymph nodes. [file jvi.00574-24-s0005.tif]

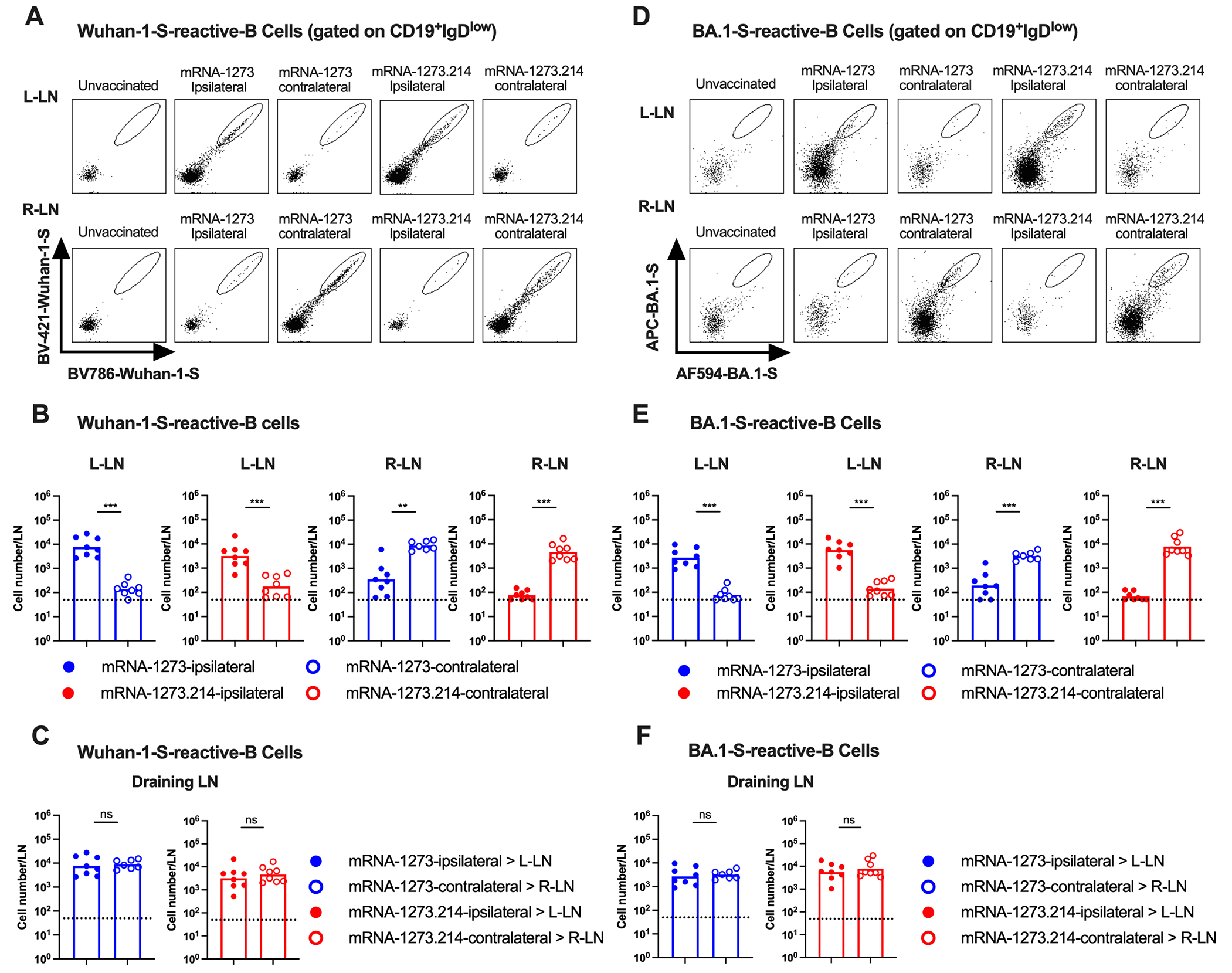

Supplement: Figure S6 — Spike-specific total B cell responses in lymph nodes. [file jvi.00574-24-s0006.tif]

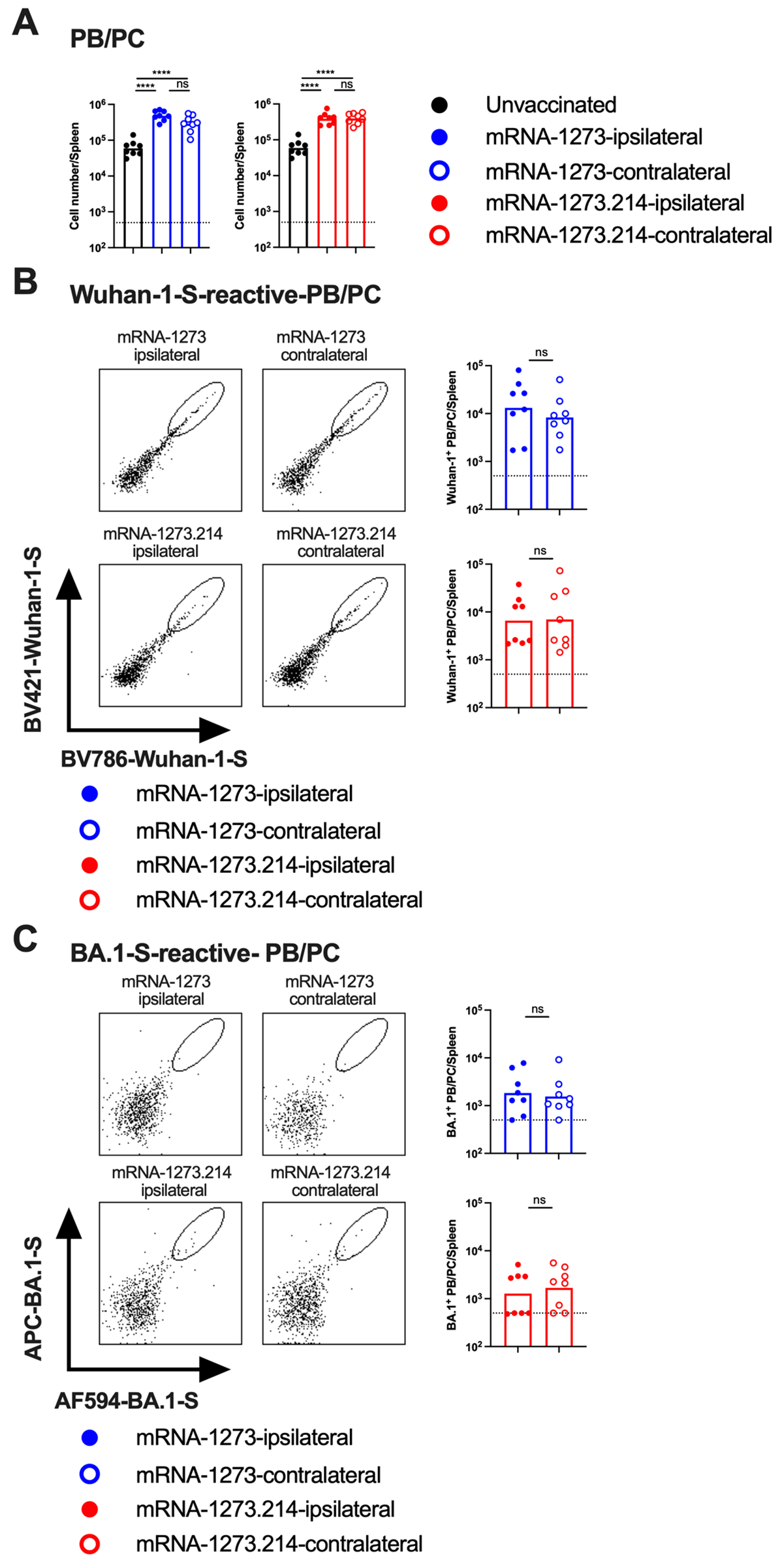

Supplement: Figure S7 — PB/PC responses in the spleen following boosting with mRNA-1273 or mRNA1273.214. [file jvi.00574-24-s0007.tif]

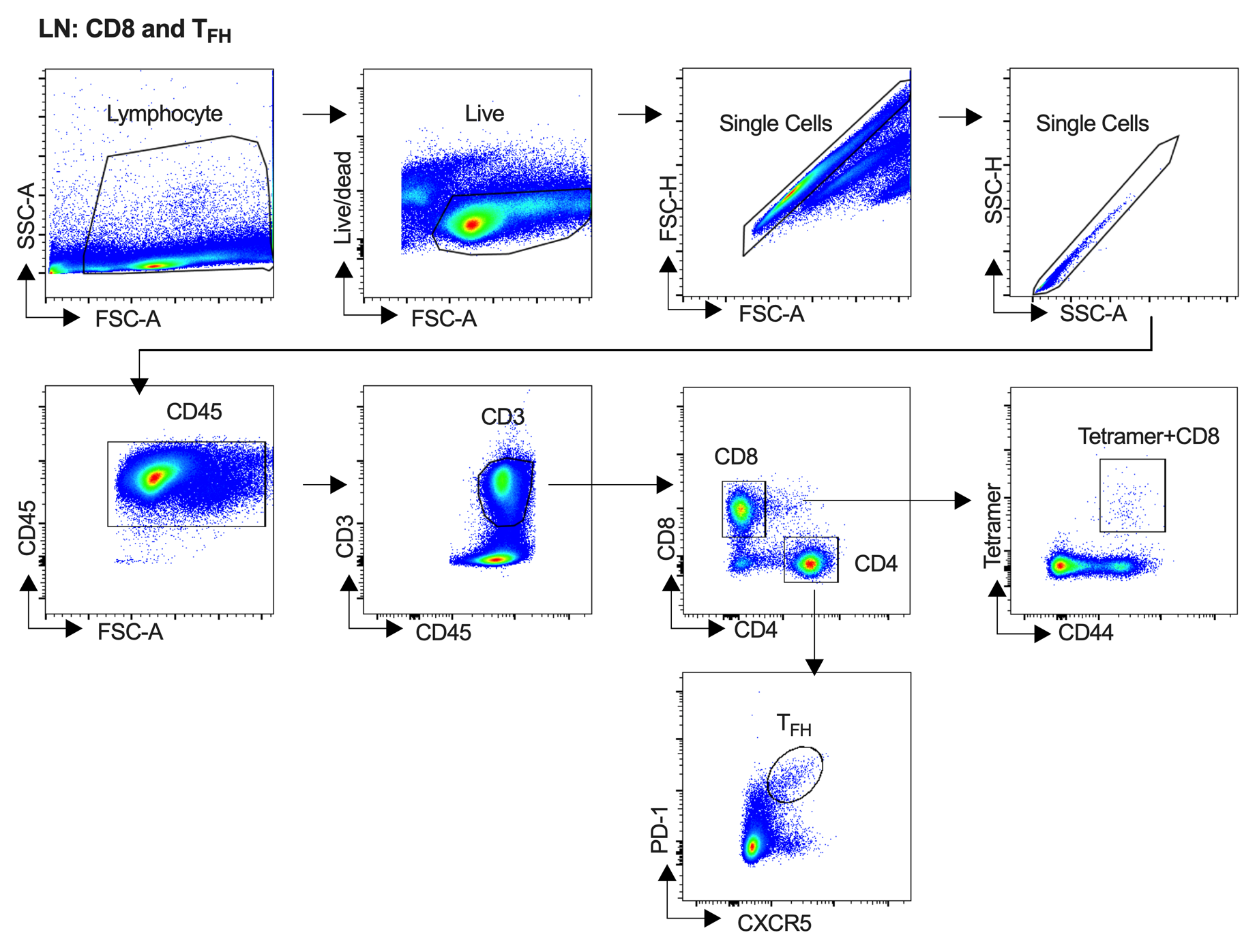

Supplement: Figure S8 — Gating strategies for analyzing spike specific CD8+ T and total TFH cells. [file jvi.00574-24-s0008.tif]

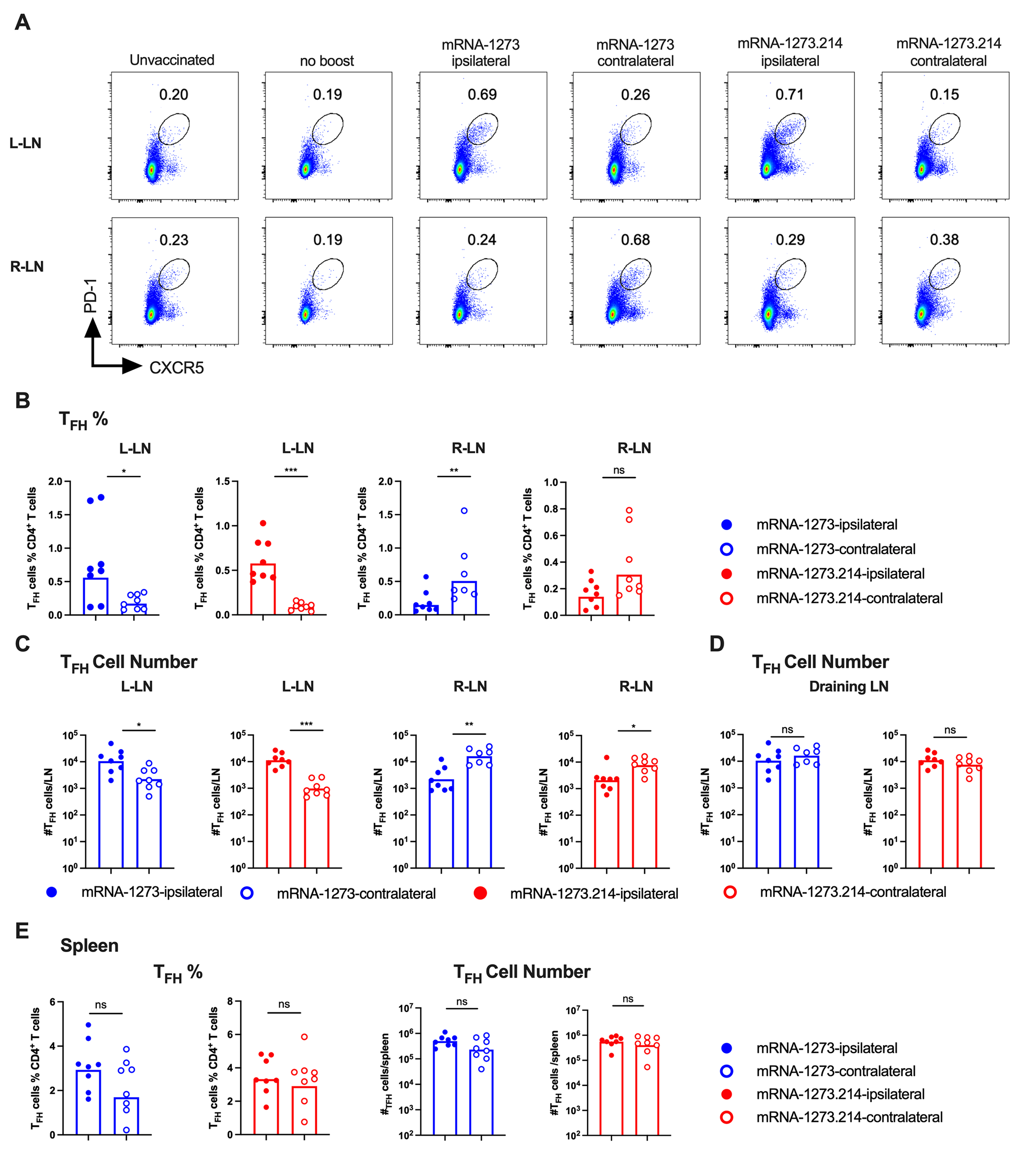

Supplement: Figure S9 — TFH cell responses in the lymph node and spleen. [file jvi.00574-24-s0009.tif]
