# Supplementary material for: Ipsilateral or contralateral boosting of mice with mRNA vaccines confers equivalent immunity and protection against a SARS-CoV-2 Omicron strain
Source: J Virol. 2024 Aug 28;98(9):e00574-24. doi: 10.1128/jvi.00574-24 (PMC11406931; doi:10.1128/jvi.00574-24)
Supplement: Supplemental figure legends — Legends for Fig. S1 to S9. [file jvi.00574-24-s0010.docx]

**­­Supplementary material for:**

**Ipsilateral or contralateral boosting of mice with mRNA vaccines confers equivalent immunity and protection against a SARS-CoV-2 Omicron strain**

Baoling Ying^1^, Chieh-Yu Liang^1^,^2^, Pritesh Desai^1^, Suzanne M. Scheaffer^1^, Sayda M. Elbashir^3^, Darin K. Edwards^3^, Larissa B. Thackray^1^, and Michael S. Diamond^1,2,4,5,6^ ¶

^1^Department of Medicine, Washington University School of Medicine, St. Louis, MO 63110, USA

^2^Department of Pathology & Immunology, Washington University School of Medicine, St. Louis, MO, USA

^3^Moderna, Inc., Cambridge MA, USA

^4^Department of Molecular Microbiology, Washington University School of Medicine, St. Louis, MO, USA

^5^The Andrew M. and Jane M. Bursky Center for Human Immunology and Immunotherapy Programs, Washington University School of Medicine. St. Louis, MO, USA

^6^Center for Vaccines and Immunity to Microbial Pathogens, Washington University School of Medicine, Saint Louis, MO, USA

Address correspondence to Michael S. Diamond: mdiamond@wustl.edu

**SUPPLEMENTAL FIGURE LEGENDS**

**Figure S1. Serum neutralization of WA1/2020 D614G and BA.1 viruses, Related to Figure 2.** Seven- to nine-week-old female K18-hACE2 transgenic mice were immunized with a primary mRNA-1273 vaccination series and then boosted in the ipsilateral or contralateral leg with mRNA-1273 or mRNA-1273.214. Neutralizing antibody responses against WA1/2020 D614G and BA.1 were assessed from serum samples from one day before (**A**) or four weeks after (**B**) booster dose vaccines (n = 15-18). Neutralization curves corresponding to individual mice are shown for the indicated vaccines. Each point represents the mean of two technical replicates.

**Figure S2**. **Flow cytometry gating strategies for B cells in the LN and spleen, Related to Figures 3 and 4, and S3-S7**. **A.** Spike-specific B cells were gated as lymphocytes (FSC-A/SSC-A), singlets (FSC-H/FSC-A), live cells (Viability dye eF506^-^), CD19^+^IgD^low^ cells, followed by Wuhan-1 spike (BV421^+^BV786^+^) or BA.1 spike (APC^+^AF594^+^) positive staining. GCBs were gated for lymphocytes (FSC-A/SSC-A), singlets (FSC-H/FSC-A), live cells (Viability dye eF506^-^), CD19^+^, IgD ^low^, Fas^+^GL7^+^, decoy Oval^-^ (non-specific binding negative), followed by Wuhan-1 spike (BV421^+^BV786^+^) or BA.1 spike (APC^+^AF594^+^). Wuhan-1 spike (BV421^+^BV786^+^) or BA.1 spike (APC^+^AF594^+^) reactive GCBs were subgated into cross-reactive or Wuhan-1 or BA.1 specific GCBs. **B.** PBs/PCs were gated for lymphocytes (FSC-A/SSC-A), singlets (FSC-H/FSC-A), live cells (Viability dye eF506^-^), CD19^+^, IgD^low^, CD138^+^TACI^+^, decoy Oval- (non-specific binding negative), followed by Wuhan-1-spike (BV421^+^BV786^+^) or BA.1 spike (APC^+^AF594^+^). Wuhan-1 spike (BV421^+^BV786^+^) or BA.1 spike (APC^+^AF594^+^) reactive PBs/PCs were subgated into cross-reactive or Wuhan-1 or BA.1 specific PBs/PCs. Note, panels **A** and **B** were derived from the same sample, and thus the initial gating steps (lymphocytes, single cells, live cells, CD19^+^ B cells, and CD19^+^IgD^low^ antigen-experienced B cells) are identical.

**Figure S3. Frequency of germinal center B cells and plasmablast/plasma cells in lymph nodes following boosting with mRNA-1273 or mRNA1273.214 vaccines, Related to Figures 3 and 4.** (**A-C**) GCBs**.** Frequency of all GCBs (**A**), Wuhan-1 spike-reactive GCBs (**B**), and BA.1 spike-reactive GCBs (**C**) among antigen-experienced B cells (CD19^+^IgD^low^). (**D-F**) PB/PCs. Frequency of all PB/PCs (**D**), Wuhan-1 spike-reactive PB/PCs (**E**), and BA.1 spike reactive PB/PCs (**F**) among antigen-experienced B cells (CD19^+^IgD^low^). Note, the number of GCBs (**A**) or PB/PCs (**D**) in the LNs on the respective side (L-LN or R-LN) from unvaccinated mice are shown in each graph with the different vaccines (mRNA-1273 or mRNA1273.214) for comparison purposes. Data are from two independent experiments (n = 7-8, each data point represents an individual mouse, column heights indicate median values). Statistical analyses: **A, D,** One-way ANOVA with Tukey’s post-test; **B-C, E-F,** unpaired two-tailed Mann-Whitney test: ns, not significant; ^∗^ p < 0.05, ^∗∗^ p < 0.01, ^∗∗∗^ p < 0.001, ^∗∗∗∗^ p < 0.0001.

**Figure S4. Virus type-specific and cross-reactive GCB responses in lymph nodes following boosting with mRNA-1273 or mRNA1273.214 vaccines, Related to Figure 3. A**. Representative flow cytometry scatter plots of BA.1-S cross-reactive and Wuhan-1 spike-specific GCBs from the total Wuhan-1 spike-reactive GCB population. **B**. Representative flow cytometry scatter plots of Wuhan-1 spike cross-reactive and BA.1 spike-specific GCBs from the total BA.1 spike-reactive GCB population. **C-D**. Total numbers of Wuhan-1 spike specific GCBs in the respective LNs (**C**) or dLN (**D**). Data in (**D**) corresponds to data in (**C**) and is replotted for direct statistical comparison. **E-F**. Total numbers of Wuhan-1/BA.1 spike cross-reactive GCBs in the respective LNs (**E**) or dLN (**F**). Data in (**F**) corresponds to data in (**E**) and is replotted for direct statistical comparison. **G-H**. Total numbers of BA.1 spike-specific GCBs in the respective LNs (**G**) or dLN (**H**). Data in (**H**) corresponds to data in (**G**) and is replotted for direct statistical comparison. Results are from two experiments (n = 7-8, each data point represents an individual mouse, column heights indicate geometric mean values, dotted lines show the LOD). Statistical analyses: **C-H,** unpaired two-tailed Mann-Whitney test: ns, not significant; ^∗^ p < 0.05, ^∗∗^ p < 0.01, ^∗∗∗^ p < 0.001.

**Figure S5. Virus type-specific and cross-reactive PB/PC responses in lymph nodes following boosting with mRNA-1273 or mRNA1273.214 vaccines, Related to Figure 4. A**. Representative flow cytometry scatter plots of BA.1 spike cross-reactive and Wuhan-1 spike-specific PBs/PCs from total Wuhan-1 spike-reactive PB/PC population. **B**. Representative flow cytometry scatter plots of Wuhan-1 spike cross-reactive and BA.1 spike-specific PBs/PCs from total BA.1 spike-reactive PBs/PCs. **C-D**. Total numbers of Wuhan-1 spike-specific PBs/PCs in the respective LNs (**C**) or dLN (**D**). Data in (**D**) corresponds to data in (**C**) and is replotted for direct statistical comparison. **E-F**. Total numbers of Wuhan-1/BA.1 spike cross-reactive PBs/PCs in the respective LNs (**E**) or dLN (**F**). Data in (**F**) corresponds to data in (**E**) and is replotted for direct statistical comparison. **G-H**. Total numbers of BA.1 spike-specific PBs/PCs in the respective LNs (**G**) or dLN (**H**). Data in (**H**) corresponds to data in (**G**) and is replotted for direct statistical comparison. Results are from two experiments (n = 7-8, each data point represents an individual mouse, column heights indicate geometric mean values, dotted lines show the LOD). Statistical analyses: **C-H,** unpaired two-tailed Mann-Whitney test: ns, not significant; ^∗^ p < 0.05, ^∗∗∗^ p < 0.001.

**Figure S6. Spike-specific total B cell responses in lymph nodes following boosting with mRNA-1273 or mRNA1273.214 vaccines, Related to Figures 3 and 4. A**. Representative flow cytometry scatter plots of Wuhan-1 spike-reactive B cells. **B**. Total numbers of Wuhan-1 spike-reactive B cells in the respective LNs. **C**. Comparison of numbers of Wuhan-1 spike-reactive B cells in the DLNs. Data in (**C**) corresponds to data in (**B**) and is replotted for direct statistical comparison. **D**. Representative flow cytometry scatter plots of BA.1 spike-reactive B cells. **E**. Total number of BA.1 spike-reactive B cells in respective LNs. **F**. Comparison of numbers of BA.1 spike-reactive B cells in the DLNs. Data in (**F**) corresponds to data in (**E**) and is replotted for direct statistical comparison. Results are from two experiments (n = 7-8, each data point represents an individual mouse, column heights indicate geometric mean values, dotted lines show the LOD). Statistical analyses: **B, C, E, F,** unpaired two-tailed Mann-Whitney test: ns, not significant; ^∗∗^ p < 0.01, ^∗∗∗^ p < 0.001.

**Figure S7. PB/PC responses in the spleen following boosting with mRNA-1273 or mRNA1273.214, Related to Figure 4.** Seven- to nine-week-old female K18-hACE2 transgenic mice were immunized with a primary mRNA-1273 vaccination series and then boosted 11 to 12 weeks later in the ipsilateral or contralateral leg with mRNA-1273 or mRNA-1273.214. Seven days after boosting, spleens were harvested and analyzed for PBs/PCs responses by flow cytometry. **A**. Total number of CD19^+^IgD^lo^CD138^+^TACI^+^ PB/PCs. Note, the number of PB/PCs from unvaccinated mice are shown in each graph with the different vaccines (mRNA-1273 or mRNA1273.214) for comparison purposes. **B**. Representative flow cytometry scatter plots (left) and quantification (right) of Wuhan-1 spike-reactive PBs/PCs. **C**. Representative flow cytometry scatter plots (left) and quantification (right) of BA.1 spike reactive PBs/PCs. Data are from two independent experiments (n = 7-8, each data point represents an individual mouse, column heights indicate geometric mean values). Statistical analyses: **A,** One-way ANOVA with Tukey’s post-test; **B-C,** unpaired two-tailed Mann-Whitney test: ns, not significant; ^∗∗∗∗^ p < 0.0001.

**Figure S8**. **Gating strategies** **for analyzing spike specific CD8^+^ T and total T_FH_ cells in the LN and spleen, Related to Figures 5 and S6.**  Cells were gated for lymphocytes (FSC-A/SSC-A), singlets (FSC-H/FSC-A; SSC-H/SSC-A), CD45^+^, CD3^+^, CD8^+^, CD4^+^, followed by tetramer^+^CD44+ (tetramer^+^CD8^+)^ and PD-1^+^CXCR5^+^ (T_FH_).

**Figure S9. T_FH_ cell responses in the lymph node and spleen following boosting with mRNA-1273 or mRNA1273.214 vaccines, Related to Figure 5.** Seven- to nine-week-old female K18-hACE2 mice were immunized with a primary two-dose vaccination series spaced three weeks apart in the left hind leg with mRNA-1273. Animals then were boosted 11 to 12 weeks later in the left (ipsilateral) or right (contralateral) leg with 1 µg of mRNA-1273 or mRNA-1273.214. Seven days after boosting, inguinal LNs from the left, right side and spleens were analyzed for T_FH_ responses by flow cytometry. **A**. Representative flow cytometry scatter plots of PD1^+^CXCR5^+^ T_FH_ cells in the LN. **B**. Frequency PD1^+^CXCR5^+^ T_FH_ cells in respective LNs. **C**. Total cell number of PD1^+^CXCR5^+^ T_FH_ cells in the DLNs. **D**. Comparison of numbers of T_FH_ cells in the DLNs. Data in (**D**) corresponds to data in (**C**) and is replotted for direct statistical comparison. **E**. Frequency and total cell number of T_FH_ cells in the spleen. Data are from two experiments (n = 7-8, each data point represents an individual mouse, column heights indicate geometric mean values). Statistical analyses: **B**, **C, D, E,** unpaired two-tailed Mann-Whitney test: ns, not significant; * p < 0.05, ^∗∗^ p < 0.01, ^∗∗∗^ p < 0.001.
